# Beyond the Battlefield: Examining Acute Kidney Injury in Yemen's Humanitarian Crisis

**DOI:** 10.1002/puh2.70308

**Published:** 2026-07-02

**Authors:** Gamal Esam Ahmed Alsakkaf

**Affiliations:** ^1^ Department of Clinical Pharmacy, Faculty of Pharmacy Near East University Nicosia North Cyprus Cyprus

**Keywords:** acute kidney injury, cholera, humanitarian crisis, malaria, renal replacement therapy, Yemen

## Abstract

**Background:**

Acute kidney injury (AKI) represents a growing yet under‐recognized public health threat in Yemen, where ongoing conflict has exacerbated vulnerabilities to infectious diseases and waterborne illnesses. Sepsis and severe volume depletion—secondary to diarrheal diseases such as cholera—are leading contributors to AKI incidence, especially in a region with a collapsing health infrastructure and limited access to dialysis.

**Objective:**

This review consolidates existing evidence on the burden, causes, and outcomes of infection‐related and dehydration‐induced AKI in Yemen. It emphasizes systemic gaps in surveillance, diagnostic capacity, and treatment and suggests practical interventions for low‐resource, conflict‐affected settings.

**Methods:**

This narrative review conducted a structured search of international databases and gray literature, including sources from humanitarian agencies and regional health authorities. Studies reporting on AKI incidence, etiologies, and healthcare access in Yemen from 2014 to 2024 were included.

**Results:**

The data indicate a disproportionate burden of AKI among children and internally displaced populations within cholera‐endemic governorates. Shortages of dialysis resources, delayed diagnoses, and underreporting collectively worsen mortality risks. Most documented AKI cases are associated with hypovolemia, systemic infections, and a deficiency in preventive measures.

**Conclusion:**

Infection‐driven and dehydration‐related AKI are critical yet preventable causes of morbidity and mortality in Yemen. Integrating AKI prevention into diarrheal disease programs, scaling up oral rehydration and sepsis management, and strengthening community health systems are essential to mitigating renal complications and saving lives.

## Graphical abstract

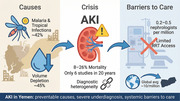

## 1 Background

Yemen's ongoing conflict has devastated infrastructure and health services. As of late 2024, only about 50% of Yemen's 5056 pre‐war health facilities remain functional, and a significant portion of the population continues to face major barriers to accessing health care [1, 2]. Basic services, such as water and electricity, are unreliable, and an estimated 19.6 million people struggle to access even the most basic health services [3]. Civil servant salaries remain largely unpaid, further reducing health system capacity [2, 4].

This systemic collapse has made Yemen a major focus of cholera in recent years [5]. Since October 2016, the country has experienced a prolonged outbreak with very high suspected case counts and substantial mortality [6]. Inadequate water, sanitation, and hygiene (WASH), together with limited access to timely treatment, remain key drivers of ongoing transmission and severe dehydration risk [2, 3, 5, 7].

Other communicable diseases, including malaria, dengue, typhoid, and hepatitis, have resurged amid the weakened health system, whereas zoonoses such as leptospirosis pose ongoing risks, especially following seasonal floods. The current context is exacerbated by insufficient funding, persistent shortages of medical staff and supplies, and damage to medical infrastructure [7, 8].

In this context of converging crises, acute kidney injury (AKI) emerges not simply as a complication of disease but as a sentinel health event. The incidence and outcomes of AKI in Yemen serve as a direct, quantifiable measure of the synergistic failure of public health systems, medical infrastructure, and environmental safety. It represents the final common pathway for many of the conflict's deadliest consequences, including severe volume depletion from diarrheal disease and systemic infection from a host of pathogens. The study of AKI in Yemen is therefore the study of a systems collapse, in which the social and political determinants of health—namely, conflict—create the precise biological conditions for organ failure while simultaneously destroying the means to prevent or treat it [9, 10].

### 1.1 Infection‐Driven AKI

Severe systemic infections, notably sepsis, contribute to renal hypoperfusion and inflammatory injury [9]. Tropical pathogens such as *Leptospira interrogans* can directly invade renal tissue, inducing interstitial nephritis and vasculitis. As documented in the literature, tropical zones support the propagation of infections that can cause AKI, including malaria, leptospirosis, HIV, and diarrheal diseases [11, 12].

### 1.2 Volume Depletion–Induced AKI

Significant hypovolemia resulting from diarrheal illnesses—including cholera, rotavirus, enterotoxigenic *Escherichia coli, and shigellosis*—leads to prerenal AKI. For example, *Vibrio cholerae* induces extensive secretory diarrhea; if untreated, severe hypovolemia rapidly precipitates renal ischemia and acute tubular injury (acute tubular necrosis [ATN]). Malnutrition related to conflict and delays in medical care further heighten the risk of severe dehydration and subsequent renal injury [13].

Limited funding, inadequate laboratory infrastructure, and unsafe conditions hinder the collection of comprehensive data on the incidence of AKI in Yemen. AKI is a common but under‐recognized complication of Yemen's public health crisis. However, systematic data on AKI incidents are scarce, and routine surveillance is nearly nonexistent. By analyzing hospital and outbreak reports, we can estimate the AKI burden and the factors that worsen it. The actual national burden is almost certainly underestimated due to the lack of surveillance and reporting systems.

## 2 Objectives

- Summarize existing data on AKI in Yemen, covering incidence, causes, age and regional patterns, and outcomes.
- Identify triggering infections such as cholera and sepsis, and local vulnerabilities including malnutrition and displaced populations.
- Point out deficiencies in AKI surveillance, diagnosis, and treatment within Yemen's resource‐limited, conflict‐affected environment.
- Contrast Yemen's AKI situation with other humanitarian crises and suggest practical interventions like rehydration programs and workforce training.

## 3 Methods

### 3.1 Design and Rationale

This is a narrative review. A formal systematic review was not undertaken because primary AKI data from Yemen are scarce, the available studies are heterogeneous in design and case definition, much of the evidence base resides in gray literature without standard reporting, and the absence of comparator data renders standardized quality‐assessment instruments inapplicable to most included sources. A narrative synthesis was therefore the most transparent way to integrate peer‐reviewed and gray‐literature evidence on AKI in this conflict‐affected setting.

### 3.2 Search Strategy

Publications and reports released between 2014 and 2024 were searched across PubMed–MEDLINE, Embase, Google Scholar, the World Health Organization (WHO) Global Index Medicus, and the Index Medicus for the Eastern Mediterranean Region (IMEMR). Gray literature was retrieved from humanitarian portals (ReliefWeb, OCHA) and institutional reports from the WHO, UNICEF, Médecins Sans Frontières (MSF), the International Society of Nephrology (ISN), and the World Bank. MeSH terms and free‐text keywords combined renal failure, disease context, and geography (e.g., “acute kidney injury” OR “acute renal failure” AND Yemen AND [conflict OR war OR humanitarian crisis OR cholera OR malaria OR malnutrition OR trauma]), supplemented by Arabic terms such as “Al‐fashal al‐kalawi al‐haad” (acute renal failure [ARF]). Targeted system‐level searches additionally retrieved ISN Global Kidney Health Atlas reports for the Middle East, audits of Yemeni dialysis and transplant capacity, and humanitarian‐ethics frameworks for renal replacement therapy (RRT) rationing in crisis settings.

### 3.3 Eligibility Criteria

#### 3.3.1 Inclusion Criteria

Sources were eligible if they (i) addressed AKI—or pre‐KDIGO ARF—incidence, etiology, severity, outcomes, management, or access to kidney‐care services in Yemen; (ii) were published between 2014 and 2024, with key earlier hospital‐based AKI series cited as foundational evidence where they directly informed the contemporary picture; and (iii) appeared in peer‐reviewed journals, indexed institutional reports, or gray‐literature sources written in English or Arabic.

#### 3.3.2 Exclusion Criteria

Sources were excluded if they (i) addressed chronic kidney disease (CKD), end‐stage renal disease, or transplantation outcomes without an AKI component; (ii) reported populations that were not Yemeni, or whose data could not be disaggregated for Yemen; or (iii) consisted of commentary, editorial, or opinion pieces without primary or secondary data.

### 3.4 Study Selection and Quality Appraisal

Records were screened by the author against the eligibility criteria, first by title and abstract and then by full text where eligibility could not be determined from the abstract alone. Several included sources are gray literature or locally published studies that, while indispensable in data‐scarce, conflict‐affected settings, vary in reporting standards and methodological rigor. No formal quality‐assessment instrument was applied because of the small number of available primary studies, the heterogeneity of designs and AKI case definitions (KDIGO‐aligned, biochemical, clinical‐ARF, or implicit), and the frequent absence of comparator groups rendered standardized appraisal tools uninformative. Each included study was instead narratively appraised on five dimensions: sample size, AKI case definition, single‐ versus multi‐center scope, indexed versus gray‐literature provenance, and external generalizability to Yemen's wider population. These appraisals informed the weight given to each finding in the synthesis, and findings derived predominantly from gray literature should be interpreted with this variability in mind.

## 4 Results

### 4.1 Yield of the Literature Search

Despite comprehensive searching across five major databases and multiple grey‐literature sources, the evidence base for AKI in Yemen is small. Approximately six primary hospital‐based AKI series spanning 1998–2024 [14, 15, 16, 17, 18, 19, 20, 21] were identified, together with a further four studies addressing related kidney‐care topics (dialysis capacity, CKD risk factors, and hemodialysis adequacy) without primary AKI data, summarized in Table 2. This scarcity of primary evidence is itself a finding: It reflects the collapse of research and reporting infrastructure in conflict zones [22] and frames the synthesis that follows.

### 4.2 AKI Incidence, Etiology, and Severity in General Cohorts

Published Yemeni evidence on AKI incidence and etiology is confined to the small number of hospital‐based series summarized in Table 1. Across more than two decades and across studies using different AKI case definitions, two etiological pathways appear repeatedly in the available series: tropical infection (principally malaria, 23%–28% of admissions) and severe volume depletion (principally diarrheal disease, including cholera) [14, 15, 16, 17, 18, 19, 20, 21]. Although the evidence base is limited and heterogeneous, these recurring patterns support a practical public‐health inference: Many episodes of community‐acquired AKI in this context may be reducible through upstream interventions (vector control, water and sanitation infrastructure, and early oral or intravenous rehydration).

**TABLE 1:** Summary of key Yemeni acute kidney injury/acute renal failure (AKI/ARF) studies.

Study (reference)	Setting (period)	Findings (AKI cases/etiology)	AKI^a^ definition/criteria	Notes
Al‐Rohani [14]	Hodeidah (1998–02)	ARF^b^ incidence 23.5/million/year; tropical infections 45% (malaria 27.9%, diarrhea 13.6%); 75.4% recovered	Clinical ARF (unstandardized)	Early diagnosis improved outcomes but hampered by delayed care/lab deficits
Al‐Rohani [15]	Sana'a (2008–09)	Tropical infections 45.3%; malaria leading; hypotension 28.6%; 39.9% required dialysis	Clinical ARF (unstandardized)	Low dialysis utilization despite severity implies limited access
Wazae Abuasba [16]	Sana'a (2015–16)	Malaria 23%; prerenal hypovolemia 45%; intrinsic (ATN) 54.5%; recovery 59%; mortality 8%	KDIGO‐aligned	KDIGO‐aligned; 54.5% intrinsic suggests late presentation
Al Sheebani et al. [20]	Hodeidah (2017)	Cholera‐associated ARF requiring dialysis; mortality 18.4%	Biochemical (serum creatinine threshold)	Extreme dehydration‐driven AKI; critical supply shortages
Alassar et al. [21]	Sana'a/Taiz (2017)	94.8% of 172 cholera inpatients severely dehydrated	Clinical dehydration grading (AKI implied, not quantified)	AKI not quantitatively reported; highlights volume depletion

Abbreviation: ATN, acute tubular necrosis.

^a^ AKI is used throughout the text as the general term unless referring specifically to a study that employed ARF criteria.

^b^ ARF = acute renal failure, the term used in older studies prior to the standardization of AKI terminology by KDIGO (Kidney Disease: Improving Global Outcomes). Where a study originally used the term ARF, it is preserved in this table for fidelity to the source.

Two later Yemeni hospital series supported the same overall pattern: AKI/ARF admissions were largely driven by tropical infection (with malaria prominent) and by hemodynamic compromise related to shock or volume depletion. Al‐Rohani reported that infection accounted for 45.3% of ARF admissions and hypotensive shock for 28.6%, but only 39.9% of severely ill ARF patients received dialysis, highlighting constrained access to RRT [15]. Wazae Abuasba, the only study applying KDIGO‐aligned criteria, described 143 AKI patients at Yemen's nephrology referral center and reported malaria in 23% of cases, prerenal hypovolemia in 45%, intrinsic ATN in 54.5%, in‐hospital recovery in 59%, and mortality in 8% [16]. The predominance of intrinsic ATN despite frequent hypovolemia is consistent with delayed presentation, where potentially reversible prerenal AKI progresses before patients reach tertiary care.

Regarding other infections beyond malaria and cholera, there are no published Yemeni series specifically addressing AKI caused by non‐cholera, non‐malaria infections. Anecdotal reports suggest that leptospirosis, associated with floods and rodent exposure, and sepsis from bacterial pneumonia or typhoid may contribute to hospital AKI cases as observed in other tropical regions. No data are currently available concerning dengue or hepatitis E in relation to AKI within Yemen. Nonetheless, given the endemic nature of malaria in specific regions and waterborne outbreaks, infection‐induced AKI is likely prevalent. Data from *Yemen Journal of Medicine* reviews underscore the evolving diagnostic and management landscape for AKI in such settings, particularly the role of novel biomarkers and point‐of‐care diagnostics that remain underutilized in Yemen's health system [23, 24].

### 4.3 Clinical Severity and Dialysis Access

The severity profile of the admitted patients highlights the crisis in access to care. In the Al‐Rohani cohort, multi‐organ failure (MOF) occurred in 19.7% of ARF cases and was accompanied by a high mortality rate (18%–26%) [15]. Critically, despite this high severity—where MOF often necessitates RRT—only 39.9% of ARF patients received dialysis. This utilization rate is disproportionately low relative to the documented clinical severity, strongly indicative of limited access to RRT.

Further data from the nephrology center in Sana'a between 2015 and 2016 reinforces the severe clinical demand [16]. Among 143 AKI patients, intrinsic renal AKI (ATN) was more frequent (54.5%) than prerenal AKI (45.4%). The dominance of established renal injury suggests delayed care, as early dehydration would typically present as reversible prerenal AKI. Furthermore, whereas 58.7% of patients recovered without dialysis, 33.5% required dialysis, which was used either peritoneal dialysis (PD) or hemodialysis (HD).

### 4.4 Dialysis and Kidney‐Care Infrastructure

The landscape of kidney‐care capacity in Yemen reflects broader regional and international assessments. The ISN Global Kidney Health Atlas reports (2021 and 2023–2024 updates) characterize the Middle East region, including Yemen, as having a severely constrained nephrology workforce, limited dialysis facility density, and minimal integration of AKI services across the continuum of care [25, 26]. As documented in recent dialysis center audits, around 28 of the pre‐war 32 dialysis centers remain operational but face critical supply shortages [27]. These facilities, located primarily in Sana'a (Al‐Thawra), Aden (Al‐Gamhouria Teaching Hospital), and regional centers in Ibb, Hodeidah, and Sa'adah, serve both CKD and emergency AKI populations, resulting in substantial resource rationing.

As summarized in Table 2, studies from individual dialysis centers document the caseload profile. A recent retrospective analysis from Al‐Thora General Hospital in Ibb Governorate (2024) examined 4194 hemodialysis patients and identified older age (>65 years), diabetes mellitus, cardiovascular disease, and cerebrovascular accident as major predictors of mortality [27]. Although primarily a CKD mortality study, this provides insight into the capacity constraints and clinical complexity of dialysis patients in operational centers. Similarly, a 2024 biochemical audit from Ibb University assessed serum urea, creatinine, and cardiac enzyme trends before and after standard HD sessions in 50 CKD patients, demonstrating approximately 51% reduction in urea and 63% reduction in creatinine—indicating reliance on conventional intermittent HD and some monitoring of dialysis adequacy [28]. These metrics are particularly relevant to AKI management, where dialysis frequency and intensity are often compromised by resource constraints.

**TABLE 2:** Related kidney‐care studies in Yemen.

Study (reference)	Setting	Findings	Notes
Predictors of mortality [27]	Ibb (2024)	Major mortality predictors: age >65, DM, CVD, stroke	Reflects rationing between ESRD and AKI
Biochemical audit [28]	Ibb (2024)	HD efficacy: urea ↓51%, creatinine ↓63%	Demonstrates dialysis monitoring capacity
Risk factors [29]	Sa'adah (2019)	Risk factors: HTN (OR 4.2), DM (OR 3.1), khat (OR 2.8), NSAIDs (OR 2.3)	Identifies upstream risk profile
Hodeidah caseload [30]	Hodeidah (2023)	560 patients: CKD 99.1%, ARF 0.89%	Selection bias; only severe AKI reaches dialysis

Abbreviations: AKI, acute kidney injury; ARF, acute renal failure; CKD, chronic kidney disease.

A case‐control study from Sa'adah Governorate (2019) examined risk factors for end‐stage renal failure among 86 ESRD cases and 263 controls at Aljomhory Hospital [29]. Hypertension (OR 4.2), diabetes (OR 3.1), khat chewing (OR 2.8), NSAID use (OR 2.3), and family history (OR 2.1) were identified as significant risk factors for progression to ESRD. Many of these same factors—particularly hypertension, diabetes, and NSAIDs—also predispose to AKI and worsen renal recovery in acute settings.

Cross‐sectional data from Hodeidah's dialysis center (2024) documented a caseload of 560 patients with chronic renal failure (99.1%) and 5 patients with ARF (0.89%) [30]. This low ARF proportion likely reflects referral and access bias rather than true population incidence, underscoring that AKI cases reach dialysis only when condition is severe and late.

### 4.5 Malaria and Other Tropical Infections

The dominance of malaria as an AKI cause in Yemen is supported by epidemiological surveillance data. A recent retrospective analysis of malaria trends in Yemen (2006–2021) documented fluctuating but persistent malaria endemicity, with significant cases in Hodeidah and southern governorates [18]. A cross‐sectional study from Bajil district in Hodeidah (2020) examined 450 schoolchildren and found uncomplicated Plasmodium falciparum malaria in 23.8% of cases, with anemia (62.4%) and underweight (48.2%) comorbidities—risk factors that amplify susceptibility to severe malaria‐associated AKI [17].

Clinical characterization of malaria severity in Aden documented that among adult patients with complicated malaria (including cerebral malaria, hepatic impairment, and renal failure) admitted to Al‐Gamhouria Teaching Hospital and private clinics, renal impairment manifested in a subset of severe cases, often accompanying hepatic dysfunction and hematologic complications [19]. The specific frequency of malaria‐associated AKI requiring dialysis was not quantified in that series but aligns with the 23%–27.9% range reported in earlier cohorts.

### 4.6 Diabetes and CKD Burden

Microalbuminuria (marker of early diabetic kidney disease) prevalence among Type 2 diabetic patients attending Al‐Thawra Hospital and Saref Medical Center in Sana'a (2021) was documented in a cross‐sectional study of 125 patients, with microalbuminuria and macroalbuminuria in 44.8% and 12%, respectively, and strong association with longer diabetes duration [31]. This high burden of early diabetic kidney disease substantially increases vulnerability to AKI from sepsis, dehydration, nephrotoxins, and surgery, mirroring global data that CKD is a major AKI risk modifier. Post‐transplant renal function was examined in a cohort of 113 renal transplant recipients in Sana'a (2014), where history of acute rejection and delayed graft function were independent predictors of post‐transplant anemia—conditions encompassing AKI episodes in the early post‐transplant period [32].

### 4.7 Cholera‐Associated AKI

More definitive data support cholera‐associated AKI, where severity is extreme. During Yemen's 2017 cholera epidemic in Hodeidah, 250 cases developed AKI requiring dialysis [20]. The prognosis for these patients was severely worsened by renal complications; among the 250 ARF cases, 46 deaths occurred, resulting in a case fatality rate (CFR) of 18.4% [20]. Patients requiring dialysis presented with severely elevated serum creatinine levels, which ranged from 4 to 14 mg/dL, with a median of 7 mg/dL. Management for this cohort utilized HD machines.

Additionally, a study involving 172 hospitalized cholera patients in Sana'a and Taiz (2017) reported that 94.8% exhibited severe dehydration, emphasizing the high initial risk for prerenal AKI [21]. This cohort profile highlights the massive volume‐depletion burden inherent in cholera outbreaks and the narrow window for early intervention (e.g., aggressive oral rehydration solution [ORS] and intravenous [IV] fluid resuscitation) before progression to established ATN and dialysis‐dependent AKI.

## 5 Discussion

**FIGURE 1:**
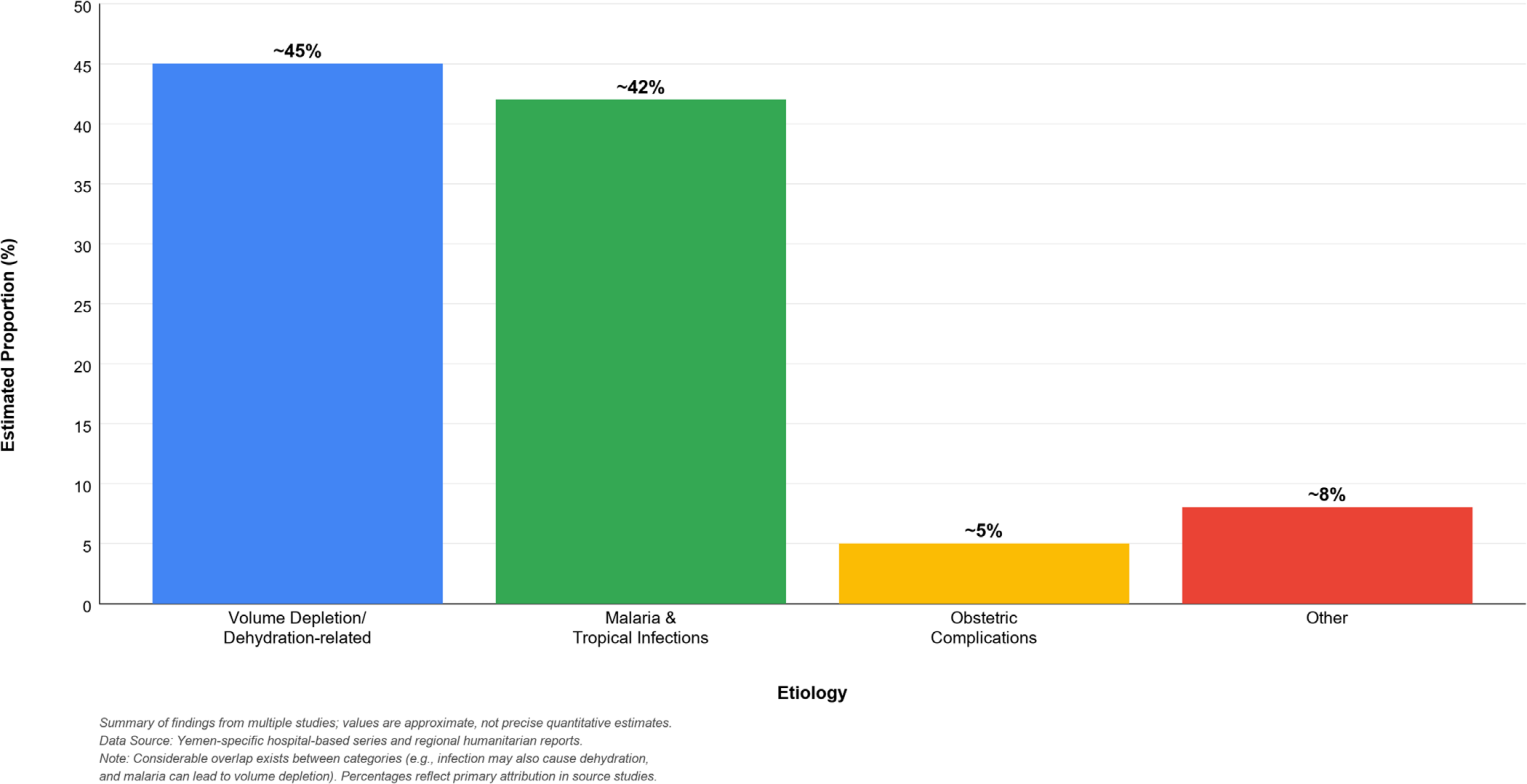
Reported causes of acute kidney injury in Yemen (2014–2024). This figure represents a qualitative summary of findings from multiple studies rather than precise quantitative estimates. Categories reflect primary attribution in source studies; overlap exists (e.g., malaria may also cause volume depletion).

Evidence from the limited published hospital series suggests that the AKI described in Yemen is predominantly community‐acquired and frequently associated with two pathways: severe infection (principally malaria) and profound volume depletion (principally cholera and diarrheal disease) [33]. In the included cohorts, these pathways account for a large proportion of reported AKI cases (Figure 1). Interpretation: In principle, many such cases could be reduced through upstream public‐health interventions (vector control, safe water and sanitation, early rehydration, and timely recognition of sepsis), although the magnitude of preventable burden cannot be quantified from the available data. More broadly, these patterns are consistent with a system‐level crisis in which conflict increases exposure to infection and dehydration while constraining access to prevention and treatment [22]. It is also likely that only a fraction of the national AKI burden reaches tertiary care; those who do are typically critically ill, and the outcomes observed in these series may therefore reflect systemic constraints as much as bedside clinical factors.

Added value of this review: Global syntheses of AKI in low‐ and middle‐income settings [34, 35, 36] and regional assessments of kidney‐care capacity [25, 26, 37] do not disaggregate Yemen, and no prior synthesis has consolidated the Yemeni evidence base. To our knowledge, this is the first decade‐spanning review (2014–2024) to integrate the sparse indexed AKI literature from Yemen with humanitarian‐agency reports and locally published audits, to map etiological burden onto the country's collapsing kidney‐care infrastructure, and to treat the data scarcity itself as a primary finding rather than a limitation. The review therefore contributes (i) a consolidated etiological profile of AKI in an active conflict setting, (ii) an explicit linkage between AKI epidemiology and the health‐system, water, and workforce gaps that produce it, and (iii) a research and policy agenda calibrated to a near‐zero baseline of routine surveillance.

Placing Yemen's AKI burden in the international context underscores the severity of this crisis. Global estimates suggest approximately 13.3 million AKI cases occur annually, with roughly 85% in low‐ and middle‐income countries (LMICs) [34]. A meta‐analysis of worldwide AKI incidence reported pooled rates of 21.6% among hospitalized adults [35]. Against these benchmarks, Yemen's reported incidence of approximately 23.5 per million per year [11] almost certainly represents a severe undercount, given that it captures only patients reaching tertiary hospitals. Hospital‐based AKI mortality in well‐resourced settings ranges from approximately 5% to 10%, whereas sub‐Saharan African hospitals report 20%–35% [35, 36]. Yemen's 8%–26% mortality range sits within the LMIC corridor, with the upper end driven by critical gaps in dialysis access.

Yemen's estimated 0.2–0.5 nephrologists per million population contrasts sharply with the global average of approximately 10 per million [25, 26, 37], placing Yemen among the most nephrology‐workforce‐deprived nations globally.

This epidemiological profile offers shared lessons with other humanitarian crises. The burden observed appears comparable to settings like the 2014–15 West Africa Ebola outbreak, where hypovolemia was the primary driver of AKI, and Haiti's 2010 cholera epidemic, which similarly overwhelmed limited renal units [38, 39, 40]. The high incidence and resulting mortality in these contexts are fundamentally attributable to the failure of basic public health functions—inadequate WASH infrastructure—coupled with the collapse of clinical capacity, including timely diagnosis and access to life‐saving RRT [41].

### 5.1 Interpreting Low Dialysis Utilization Rates

It is crucial to interpret the reported low dialysis utilization rates (e.g., 39.9% in the Al‐Rohani [15] cohort) in the context of severe clinical necessity. The patient cohorts reaching referral centers are not experiencing mild, readily reversible AKI. The severity of illness in admitted patients is profound: MOF, a condition where supportive RRT is often mandated for survival [15]. This pattern is consistent with delayed access to care, in which potentially reversible prerenal injury (e.g., from severe dehydration during diarrheal illness) progresses to established ATN before patients reach referral centers. Taken together, the available data suggest that low dialysis utilization likely reflects constrained access to RRT relative to clinical need in these settings [42].

### 5.2 Systemic Barriers to Dialysis and RRT Modality Selection

The persistent failure to provide adequate dialysis services is rooted in systemic structural collapse. Reports consistently highlight that operational centers are hampered by dysfunctional equipment and unpaid staff, facing ongoing shortages of crucial supplies like dialysis fluid and filters, leading to necessary treatment rationing [42]. The specific modality used for RRT in these settings also warrants scrutiny. Although one study noted that 33.5% of patients required dialysis with either PD or HD [16], most reports, such as those detailing the 2017 cholera outbreak, primarily describe or imply HD protocols. The overall lack of detailed information on PD utilization in many major hospital series limits our ability to assess whether PD—a modality often considered more resource‐appropriate and flexible in low‐resource and conflict‐affected settings—is being effectively implemented or if its supply chains have also failed alongside the HD infrastructure. Furthermore, barriers such as traversing checkpoints and fuel shortages impose additional burdens, preventing patients from adhering to life‐sustaining treatment schedules.

### 5.3 Humanitarian Ethics and Kidney Care in Crisis Settings

The ethical framework for dialysis and kidney‐care provision in humanitarian emergencies has received recent international attention [43]. A 2024 paper on “Ethics in humanitarian settings—relevance and consequences for dialysis and kidney care” emphasizes the necessity of transparent triage protocols, advanced communication with patients and families about resource constraints, and systematic documentation of rationing decisions [43]. Yemen's situation exemplifies the tension between clinical need and system capacity: AKI patients with MOF and severe hyperkalemia present absolute indications for dialysis, yet dialysis slots are rationed. This reality demands that Yemen's dialysis centers and humanitarian partners establish ethical frameworks for allocation, ensuring that decisions reflect clinical urgency, likelihood of renal recovery, and patients’ informed preferences.

Similarly, RRT for refugees and internally displaced populations with end‐stage kidney disease presents additional complexity [44]. Yemen's estimated 2 million internally displaced persons (IDPs) face exceptional barriers to dialysis access, including disrupted documentation, lack of a fixed address, and inability to sustain regular clinic attendance. Gray literature and humanitarian agency reports indicate that some dialysis centers prioritize IDPs with acute treatable AKI over chronic ESRD patients—a rational triage choice when capacity is severely limited—but this dynamic remains under‐documented in peer‐reviewed literature.

### 5.4 System‐Level Kidney‐Care Capacity

The ISN Global Kidney Health Atlas characterizations of the Middle East region identify Yemen as having one of the lowest nephrology workforce densities (estimated 0.2–0.5 nephrologists per million population) and minimal kidney‐care integration across primary, secondary, and tertiary levels [25, 26]. Fragmented data from individual centers demonstrate the caseload burden: Ibb's dialysis center manages predominantly ESRD patients with high comorbid disease (diabetes, hypertension, CVD) [27], whereas smaller centers in Hodeidah and Sa'adah lack intensive care infrastructure to manage critically ill AKI patients requiring vasopressor support or CRRT (continuous RRT). The *Yemen Journal of Medicine* has published clinical reviews on CRRT modalities and indications, yet few Yemeni centers possess the equipment or trained staff to deploy these therapies [24].

### 5.5 Implications and Research Agenda

Three implications follow directly from the synthesis. First, the largest mortality reductions in Yemen's AKI burden are likely to come from upstream public‐health investments—WASH infrastructure, vector control, and decentralized oral and intravenous rehydration capacity—rather than from expanding advanced nephrology services alone. Second, the absence of routine surveillance means that the true national burden cannot be quantified; a minimum‐dataset Yemeni AKI registry, anchored at functioning dialysis centers and using KDIGO‐aligned criteria, is a feasible next step that would also enable comparison with regional and global benchmarks. Third, given the fragility of hemodialysis supply chains in active conflict, PD warrants explicit evaluation as a complementary, low‐resource modality. A pragmatic research agenda would therefore include (i) point‐of‐care creatinine deployment in cholera and diarrhea treatment centers; (ii) prospective, KDIGO‐aligned registries linked to humanitarian‐agency reporting; (iii) capacity assessments and field trials of PD as an AKI‐rescue modality; and (iv) integration of AKI indicators into existing communicable‐disease surveillance.

## 6 Conclusion

In Yemen's humanitarian emergency, AKI is an important complication of infection and dehydration, but the available evidence remains limited and heterogeneous. Recurrent cholera and other diarrheal outbreaks—together with endemic infections—are consistently associated with community‐acquired AKI in the published series. Available cohort data indicate that a minority of cholera patients develop severe, dialysis‐requiring AKI, with high mortality when timely rehydration and renal‐replacement capacity are unavailable. These findings are consistent with a health‐system shock in which disruptions to WASH infrastructure and to clinical services amplify the risk and severity of AKI.

Urgent action is needed to integrate AKI prevention and treatment into existing public health efforts:

- Scale up community oral rehydration by distributing ORS and zinc through health posts and volunteers, and train community health workers to treat early dehydration to prevent AKI, as proven lifesaving in Yemen cholera waves.
- Rehabilitate water and sanitation infrastructure quickly by repairing boreholes, chlorination points, and sewage systems. Increase international WASH funding (e.g., UNICEF‐led projects) to match disease burden.
- Integrate serum creatinine testing into triage at cholera/diarrhea centers, using point‐of‐care meters to identify high‐risk patients needing transfer. Train clinicians to recognize AKI symptoms.
- Ensure all diarrhea treatment centers remain open and stocked with IV fluids and electrolytes to prevent renal injury.
- Protect dialysis services by repairing units, providing supplies, and using mobile vans in remote areas. Facilitate ambulance passages for treatment adherence.
- Add AKI cases to disease surveillance reports and equip agencies and authorities with real‐time AKI data to better allocate resources.

## 7 Limitations

As noted in the Methods, the small number of available primary publications constrains the generalizability of the findings. This review is subject to several limitations that warrant consideration. First, the ongoing conflict in Yemen and the collapse of its healthcare infrastructure have led to substantial underreporting of AKI, particularly in rural and conflict‐affected regions where diagnostic and reporting capacities are minimal. Second, the review's reliance on gray literature—such as reports from humanitarian agencies and national health bodies—introduces variability in data quality and methodological rigor. Third, most available sources lack detailed stratification of AKI etiologies, making it difficult to delineate the contributions of specific infectious pathogens or distinguish between prerenal and intrinsic forms of AKI. Fourth, selection bias is likely, as data tend to overrepresent accessible urban centers (Sana'a, Aden) while underrepresenting marginalized populations in displaced or inaccessible areas. Fifth, a substantial limitation of this review stems from diagnostic heterogeneity within the available primary data. Many of the fundamental hospital‐based series utilized the older terminology of ARF, defined primarily by clinical deterioration and biochemical measures, often without the systematic application of modern, standardized classification criteria (e.g., KDIGO or RIFLE). Finally, the cross‐sectional nature of most datasets precludes meaningful longitudinal analysis, constraining efforts to establish causality between infectious disease burden, dehydration episodes, and renal outcomes. Additionally, the reliance on grey literature and locally published studies—while unavoidable given the scarcity of indexed research from Yemen—introduces variability in reporting standards and methodological rigor.

The inability to apply a uniform AKI definition across studies is a critical constraint. Only Wazae Abuasba employed KDIGO‐aligned criteria [16]; Al‐Rohani used clinical ARF terminology without standardized staging [14, 15]; Al Sheebani et al. relied on biochemical thresholds (serum creatinine) [20]; and Alassar et al. used clinical dehydration grading, with AKI implied but not quantitatively reported [21]. These varying case definitions mean that pooled findings should be interpreted with caution.

## Author Contributions

Gamal Esam Ahmed Alsakkaf drafted the manuscript. The author reviewed and approved the final version of the manuscript. The author has read and approved the final version of the manuscript. The author had full access to all the data in this study and takes complete responsibility for the integrity of the data and the accuracy of the data analysis.

## Funding

The author has nothing to report.

## Disclosure

The author confirms that the absence of financial support had no role in the design of this critical narrative review, the search, collection, analysis, or interpretation of the evidence, the writing of the manuscript, or the decision to submit the article for publication.

## Ethics Statement

The study did not require ethics approval from an institutional review board, as it utilized existing data for retrospective analysis.

## Conflicts of Interest

The author declares no conflicts of interest.

## Transparency Statement

Gamal EA Alsakkaf affirms that this manuscript is an honest, accurate, and transparent account of the study being reported; that no important aspects of the study have been omitted; and that any discrepancies from the study as planned and, if relevant, registered, have been explained.

## Data Availability

The data that support the findings of this study are available from the corresponding author upon reasonable request.
